# Novel Lytic Bacteriophage PAT-A: Isolation, Characterization, Genome Analysis, and Biocontrol Potential Against *Agrobacterium tumefaciens*

**DOI:** 10.3390/microorganisms14010223

**Published:** 2026-01-18

**Authors:** Chenglin Liang, Wei Tian, Jianlong Liu, Zan Zhang, Dingli Li

**Affiliations:** 1 Haidu College, Qingdao Agricultural University, Laiyang 265200, China; tw17616226889@163.com (W.T.); zhangzan0919@163.com (Z.Z.); 2 College of Horticulture, Qingdao Agricultural University, Qingdao 266109, China; 201901068@qau.edu.cn (J.L.); lidingli@qau.edu.cn (D.L.)

**Keywords:** *Agrobacterium tumefaciens*, bacteriophage, phage characterization, genomic analysis, biocontrol

## Abstract

*Agrobacterium tumefaciens*, a destructive pathogen causing crown gall disease, results in substantial agricultural losses. Traditional chemical and existing biocontrol methods are limited by environmental pollution, pesticide resistance, and low efficacy, while bacteriophages emerge as a promising alternative due to their high host specificity, environmental compatibility, and low resistance risk. In this study, we isolated and characterized a lytic phage (PAT-A) targeting *A. tumefaciens*, evaluating its biological traits, genomic features, and biocontrol potential. The host strain *A. tumefaciens* CL-1 was isolated from cherry crown gall tissue and identified by 16S rDNA sequencing. Phage PAT-A was recovered from orchard soil via the double-layer agar method, showing a tadpole-shaped morphology (60 nm head diameter, 30 nm tail length) under transmission electron microscopy (TEM). Nucleic acid analysis confirmed a double-stranded DNA genome, susceptible to DNase I but resistant to RNase A and Mung Bean Nuclease. PAT-A exhibited an optimal MOI of 0.01, tolerated wide pH and temperature ranges, but was sensitive to UV (titer declined after 15 min of irradiation) and chloroform (8% survival at a 5% concentration). Whole-genome sequencing revealed a 44,828 bp genome with a compact structure, and phylogenetic/collinearity analyses placed it in the *Atuphduvirus* genus (Autographiviridae). Biocontrol experiments on tobacco plants demonstrated that PAT-A significantly reduced crown gall incidence. Specifically, simultaneous inoculation of PAT-A and *A. tumefaciens* CL-1 resulted in the lowest tumor incidence (12.0%), while pre-inoculation of PAT-A 2 days before pathogen exposure achieved an incidence rate of 33.3%. In conclusion, PAT-A is a novel strictly lytic phage with favorable biological properties and potent biocontrol efficacy against *A. tumefaciens*, enriching phage resources for crown gall management and supporting phage-based agricultural biocontrol strategies.

## 1. Introduction

*Agrobacterium tumefaciens* is a Gram-negative soil bacterium, belonging to the family Rhizobiaceae. *A. tumefaciens* infects plant roots, stems, and other parts, transferring and integrating T-DNA fragments from its Ti plasmid into the plant cell genome [1,2]. Based on this, *A. tumefaciens* has become a natural tool in genetic engineering, and the study of its T-DNA transfer system has provided important support for the development of plant genetic engineering technology [3]. This exogenous DNA can induce abnormal proliferation and division of plant cells, forming cancerous tumors (crown galls) of varying sizes and irregular shapes [4]. The formation of tumors can destroy the plant’s epidermal and cortex protective barriers, making the plant more susceptible to secondary infection by other pathogens in the soil.

*A. tumefaciens* has a broad host range (including fruit trees, flowers, and forestry plants) [2]. The pathogen mainly spread through rainwater, irrigation water, agricultural operations, and underground pests, and could invade plants through wounds [5]. The pathogen can survive in the soil or within tumor tissues for an extended period (up to several years), becoming a persistent source of infection that poses a continuous threat to surrounding healthy plants. In addition, diseased plants sold as seedlings in nurseries can spread diseases, posing a long-term threat to the healthy development of the cherry industry and increasing production costs and economic losses.

Traditional chemical control methods have issues such as environmental pollution, pesticide residues, and resistance. In the field of biological control, the screening and action mechanisms of biocontrol strains have been the focus of research in recent years. The most widely used biocontrol bacterium is the *A. radiobacter* K84 strain, which inhibits the growth of pathogenic *A. tumefaciens* by producing agrocin 84 [5,6]. However, drug-resistant strains have already appeared in some areas. Therefore, researchers continue to screen for new efficient biocontrol strains, such as certain *Pseudomonas*, *Bacillus*, and actinomycetes strains isolated from the rhizosphere soil of healthy plants [7,8]. These strains exerted their control effects through various mechanisms, including producing antibiotics, competing for ecological niches, and inducing systemic resistance in plants. Sedzicki et al. elucidated the uncharacterized structure and tyrosine-linked oligosaccharide intermediate-based catalytic mechanism of *A. tumefaciens* cyclic glucan synthase (Cgs, the enzyme synthesizing host interaction-critical cyclic β-1,2-glucan) via cryo-EM and functional analyses, providing an innovative molecular basis for targeted *A. tumefaciens* control [9]. Furthermore, certain scholars have carried out investigations on the chemoreceptors in *A. tumefaciens*. The findings indicated that the cytoplasmic chemoreceptor Atu1027 of *A. tumefaciens* C58 serves as an aerotaxis receptor, which influences the chemotaxis of agrobacteria and the invasion of *A. tumefaciens* into its host [10].

Bacteriophages are a type of virus that specifically infect bacteria, exhibiting strict host specificity [11,12]. They can recognize and invade specific types of bacteria, utilizing the bacterial metabolic system for their own replication and proliferation, ultimately leading to the lysis and death of the host bacteria [13]. Bacteriophages are widely distributed in nature and can be found in almost all environments where bacteria exist, such as soil, water, and animal intestines [14]. In the agricultural domain, bacteriophages can be employed to prevent and manage bacterial diseases in crops, thereby reducing the utilization of chemical pesticides. *Clavibacter michiganensis* subsp. *michiganensis* (Cmm) and Cmm-specific lytic bacteriophages were isolated from the rhizosphere soil and phyllospheres of diseased tomato plants, and the biocontrol potential of these bacteriophages was investigated [15]. Skliros et al. explored a novel genus of *Pseudomonas* phages and examined its biocontrol potential against *Pseudomonas syringae* pv. tomato (Pst) by leveraging its lytic characteristics and the ability to initiate the immune response of plants [11]. Additionally, bacteriophages also present potential for the biocontrol of bacterial wilt [13].

Bacteriophages, possess advantages including high host specificity and environmental compatibility. They have shown great potential in the biological control of plant diseases. In this study, *A. tumefaciens* bacteriophages were isolated from soil, and their biological characteristics, genomic features, and biocontrol effects were analyzed. This work enriches the biological information database of *A. tumefaciens* bacteriophages and provides a theoretical foundation for their application in agricultural production.

## 2. Materials and Methods

### 2.1. Plant Materials

Tobacco (*Nicotiana tabacum* L.) seeds were obtained from the Horticulture Laboratory of Haidu College, Qingdao Agricultural University, Yantai, China. The seeds were surface-sterilized with 75% ethanol for 30 s and 1% sodium hypochlorite for 5 min, followed by three rinses with sterile distilled water. Sterilized seeds were sown into plastic pots (8 cm × 8 cm) filled with sterile nutrient medium (peat moss:vermiculite:perlite = 2:1:1, *v*/*v*/*v*) and placed in a light incubator (GDN-1000D-4, Southeast instruments Co., Ltd., Ningbo, China) at 22 °C under a light cycle of 16 h of light (photosynthetic photon flux density = 200 μmol m^−2^ s^−1^) and 8 h of darkness. Tobacco plants were cultivated for 3 weeks until they reached the 4–5 true leaf stage for biocontrol experiments.

### 2.2. Bacterial Strain and Culture Conditions

*A. tumefaciens* CL-1 was isolated from the crown gall tissue of infected cherry trees (*Prunus avium* L.) in Yantai City, Shandong Province, China, and preserved by the Horticulture Laboratory of Haidu College, Qingdao Agricultural University, Yantai, China. The bacterial strain was incubated at 28 °C in a lysogeny broth (LB) or on LB agar (Changde BKMAM Biotechnology Co., Ltd., Changsha, China).

### 2.3. 16S rDNA Strain Identification of A. tumefaciens CL-1

Total DNA of *A. tumefaciens* CL-1 was extracted using the bacterial genomic DNA extraction kit (DP302-02, Tiangen Biotech Co., Ltd., Beijing, China) following the manufacturer’s instructions. PCR amplification was performed using universal primers for bacterial 16S rDNA sequencing: forward primer F (5′-TACGGYTACCTTGTTACGACTT-3′) and reverse primer R (5′-AGAGTTTGATCCTGGCTCAG-3′). The PCR conditions were as follows: 94 °C for 10 min; 35 cycles of 94 °C for 30 s, 53 °C for 30 s and 72 °C for 1 min; 72 °C for 5 min. The amplified products were verified by 1% agarose gel electrophoresis, purified using the DNA Gel Extraction Kit (DP209-02, Tiangen Biotech Co., Ltd.), and sent to Suzhou Genomics Co., Ltd. (Suzhou, China) for sequencing. BLASTn (https://blast.ncbi.nlm.nih.gov/Blast.cgi, accessed on 28 December 2025) analysis was performed to compare with known sequences. A phylogenetic tree was constructed using MEGA 11 software (version 11.0.13).

### 2.4. Bacteriophage Isolation and Purification

The bacteriophages described in this study were isolated from soil samples collected in May 2024 at the fruit orchard of Haidu College, Qingdao Agricultural University, Yantai City, China (E 120°43′48″, N 36°59′24″). The soil samples were first mixed with sterilized PBS and shaken at 160 rpm for 2 h at 28 °C. The mixture was allowed to stand for 30 min, and the supernatant was filtered through qualitative filter paper to remove large particles, followed by filtration through a 0.22 μm filter membrane (Millex-GP, Merck KGaA, Darmstadt, Germany) to remove bacterial cells and debris. The filtrate was stored at 4 °C as the phage stock solution.

For phage enrichment, 100 μL of the phage stock solution was added to 2 mL of LB containing *A. tumefaciens* CL-1 (OD_600_ = 0.5) and incubated at 28 °C with shaking at 160 rpm for 24 h. The culture was centrifuged at 8000× *g* for 10 min at 4 °C, and the supernatant was filtered through a 0.22 μm filter membrane to obtain the enriched phage solution. Phage purification was performed using the double-layer agar overlay method [16]. Briefly, 100 µL of the enriched phage solution was serially diluted (10^−1^ to 10^−6^) with sterile PBS. Each dilution (100 µL) was mixed with 100 µL of *A. tumefaciens* CL-1 (OD_600_ = 0.5) and incubated at 28 °C for 30 min to allow phage adsorption. The mixture was added to 5 mL of molten LB top agar (0.7% agar, cooled to 45 °C) and poured onto LB bottom agar plates (1.5% agar). Plates were incubated at 28 °C for 24 h, and individual transparent plaques were picked with a sterile toothpick and suspended in 1 mL of sterile phage buffer (10 mmol L^−1^ Tris-HCl, 10 mmol L^−1^ MgSO_4_∙7H_2_O, pH 7.5). This purification process was repeated three times until plaques of uniform morphology and size were obtained. The purified phage PAT-A was stored at 4 °C for subsequent experiments.

### 2.5. Transmission Electron Microscopy (TEM)

The TEM observation was performed following the method of Lu et al. (2003) [17], with modifications. The purified bacteriophage lysate from the phage-bacteria culture supernatant filtered through a 0.22 μm filter membrane was recovered. The 20 μL high-concentration suspension PAT-A was dropped onto a copper grid, then was stained with 2% phosphotungstic acid (PTA) for 10 min. The filter paper was used to absorb the stain from the side, dry it, and observe it under a transmission electron microscope (HT7700, Hitachi High-Tech Corporation, Tokyo, Japan) at an accelerating voltage of 80 kV on a carbon-coated grid.

To observe the interaction between phage PAT-A and *A. tumefaciens* CL-1, *A. tumefaciens* CL-1 (OD_600_ = 0.5) was infected with phage PAT-A at an MOI of 0.01 and incubated at 28 °C with shaking at 160 rpm for 8 h. Bacterial cells were collected by centrifugation at 5000× *g* for 5 min at 4 °C, fixed with 2.5% glutaraldehyde in PBS at 4 °C for 24 h, and then post-fixed with 1% osmium tetroxide for 2 h. The samples were dehydrated through a graded ethanol series (30%, 50%, 70%, 80%, 90%, 95%, and 100%), embedded in epoxy resin, and sectioned into ultra-thin slices (70 nm). The slices were stained with uranyl acetate and lead citrate, and observed under the TEM at 80 kV.

### 2.6. Bacteriophage Nucleic Acid Type Identification

Phage genomic nucleic acid was extracted using the TaKaRa MiniBEST Viral RNA/DNA Extraction Kit Ver.5.0 (9766, Takara Biotechnology Co., Ltd., Dalian, China) following the manufacturer’s protocol. The extracted nucleic acid was divided into four aliquots: one aliquot was used as the control, and the other three were treated with DNase I (5 U μL^−1^, 2270 A, Takara Biotechnology Co., Ltd., Dalian, China), RNase A (10 mg mL^−1^, 2158, Takara Biotechnology Co., Ltd.), and Mung Bean Nuclease (40 U μL^−1^, 2420 A, Takara Biotechnology Co., Ltd.), respectively. The reaction system (50 µL) contained 30 µL of nucleic acid sample, 2 µL of DNase I, 5 µL of 10× DNase I buffer, and 13 µL of sterile distilled water. The mixtures were incubated at 37 °C for 30 min, and the reaction was terminated by heating at 80 °C for 2 min. The reaction system (25 µL) contained 20 µL of nucleic acid sample, 0.5 µL of RNase A, and 4.5 µL of sterile distilled water. The mixtures were incubated at 37 °C for 1 h. The reaction system (50 µL) contained 30 µL of nucleic acid sample, 0.5 µL of Mung Bean Nuclease, 5 µL of 10× Mung Bean buffer, and 14.5 µL of sterile distilled water. The mixtures were incubated at 37 °C for 10 min. The digested products were analyzed by 1% agarose gel electrophoresis (120 V, 30 min) and visualized using an invitrogen iBright FL1500 imaging system (Thermo Fisher Scientific, Waltham, MA, USA).

### 2.7. Determination of Optimal Multiplicity of Infection (MOI) of Phage PAT-A

The concentration of bacterial culture and phage titer was determined separately. The phage was mixed with *A. tumefaciens* CL-1 bacterial culture at ratios of 0.001, 0.01, 0.1, 1, 10, and 100 according to the multiplicity of infection (MOI). They were incubated in a 28 °C incubator for 30 min in static conditions, then were placed on a shaker at 160 rpm/min for 8 h of shaking culture. After the incubation, they were removed, centrifuged at 4 °C, 2500× *g* for 1 min. The supernatant was filtered through a 0.22 μm filter membrane to determine the titer. The operation was conducted three times and the average value was calculated. The MOI with the highest phage titer was considered the optimal multiplicity of infection.

### 2.8. One-Step Growth Experiment

One-step growth experiment was conducted referring to the method of Cong et al. (2024) [18], with slight modifications. According to the optimal multiplicity of infection ratio, bacteriophages were mixed with the logarithmic phase *A. tumefaciens* CL-1 bacterial culture, placed in a 28 °C, 160 rpm min^−1^ shaking culture, and samples were taken at time points of 0, 30, 60, 90, 120, 150, 180, 240, 300, 360, 420, 480, and 540 min. After filtration through a filter membrane, the titer of bacteriophages was determined, and thereby a one-step growth curve of bacteriophages was plotted.

### 2.9. Characterization of pH and Temperature Tolerance

The culture medium was adjusted to corresponding pH values of 4.0, 5.0, 6.0, 7.0, 8.0, 9.0, 10.0 and 12.0, respectively, sterilized and prepared for use. The 900 μL of LB with different pH values were taken into 1.5 mL EP tubes, and 100 μL of bacteriophage (10^8^ PFU mL^−1^) was added to each tube. These tubes were incubated at 28 °C in a water bath for 60 min, and their titer was determined using the double-layer agar plate method to evaluate the pH stability of the bacteriophage.

LB liquid medium with 900 μL was taken into a 1.5 mL EP tube, and the EP tubes were placed in a water bath at 30, 40, 50, 60 and 70 °C for preheating, while three parallel experiments were set up. After the temperature of the medium stabilized, 100 μL of phage stock solution (10^8^ PFU mL^−1^) was added, and after 60 min, the EP tube was removed from the water bath and cooled on ice. A double-layer plate method was used to obtain phage titers under each condition, thereby the thermal stability of the phage was evaluated.

### 2.10. Ultraviolet Light and Chloroform Effects on PAT-A

The 2 mL of bacteriophage (10^8^ PFU mL^−1^) was taken and placed in a 35 mm sterile Petri dish. After being irradiated under an ultraviolet lamp (30 W) at a distance of 30 cm for 0, 15, 30, 45, 60, 75, 90, 105 and 120 min, the titer of bacteriophage was determined using a double-layer plate method to evaluate the sensitivity of bacteriophage to ultraviolet light.

The phage supernatant (10^8^ PFU mL^−1^) was mixed with chloroform in ratios of 0, 1%, 2%, 3%, 4% and 5% (*v*/*v*), respectively, incubated at 37 °C for 30 min, and 100 μL of the upper layer from each separated liquid was taken to determine the phage titer, so as to evaluate the sensitivity of the phage to chloroform.

### 2.11. DNA Extraction, Whole-Genome Sequencing, and Bioinformatic Analysis of PAT-A

Genomic DNA of PAT-A was extracted using Viral Genome DNA/RNA Rapid Extraction Kit (DP315-F, Tiangen Biotech Co., Ltd., Beijing, China), following the manufacturer’s protocol. The genomic DNA of phage PAT-A was sequenced using the Illumina NovaSeq conducted by Genewiz (Azenta Life Sciences, Suzhou, China). Based on the data after quality control, Velvet (version 1.2.10) was used to perform assembly using different kmer values (95, 111, 115, 121, 127). The assembly result that received the most support from all the resulting assemblies was chosen as the final assembled sequence. Based on the predicted protein sequence of the coding gene, BLAST (version 2.3.0) and other alignment software were used to compare with protein sequences in databases, and the optimal matching result was selected as the annotation for the gene. The physical termini of the phage genome were predicted using the tool PhageTerm(version 1.0) [19]. Based on the analytical results of PhageTerm, the genome sequence was reoriented, with the starting position of the sequence set at the predicted terminal cleavage site. The online PROKSEE website (https://proksee.ca, accessed on 29 December 2025) was used to calculate the GC content and GC skewness (with a sliding window of 500 bp) of PAT-A genes and an PAT-A genome circle diagram was then drawn. The ViPTree (https://www.genome.jp/viptree/, accessed on 29 December 2025) [20] was used for PAT-A genome collinearity analysis and phylogenetic tree construction.

### 2.12. Biocontrol Experiment Design of Phage PAT-A

Referring to the bacterial inoculation method by Liang et al. (2020) [21], single colonies of *A. tumefaciens* CL-1 were selected and inoculated into LB liquid medium, followed by shaking culture for approximately 16 h to prepare bacterial suspensions. Phage PAT-A was diluted to 10^8^ PFU mL^−1^ with sterile PBS. To ensure inoculation and promote uniform disease development, artificial wounding was applied. A 3 cm wound was inflicted on the stem of tobacco plants. To investigate the biocontrol efficacy, a total of six groups were established: Group 1, cutting wounds + inoculating sterile distilled water (10 µL), 0 d; Group 2, cutting wounds, 0 d → inoculating *A. tumefaciens* CL-1 (10 µL), 2 d; Group 3, cutting wounds, 0 d → inoculating *A. tumefaciens* CL-1 (10 µL), 2 d → inoculating phage PAT-A (10 µL), 4 d; Group 4, cutting wounds + inoculating phage PAT-A (10 µL), 0 d; Group 5, cutting wounds + inoculating phage PAT-A (10 µL), 0 d → inoculating *A. tumefaciens* CL-1 (10 µL), 2 d; Group 6, cutting wounds, 0 d → inoculating *A. tumefaciens* CL-1 (10 µL) + phage PAT-A (10 µL), 2 d. A total of six treatment groups were established with 30 plants per group. Infected plants were cultivated further in the illumination incubator under the same conditions as described in Section 2.1. The tumor incidence and development were recorded. Tumor incidence rates in different groups were counted and calculated on the 18th day after the first treatment. Tumor incidence was calculated as (number of plants with tumors/total number of plants) × 100%. For each group, three independent biological replications were conducted.

### 2.13. Statistical Analysis

Statistical analysis were performed with IBM SPSS Statistics (Version 23.0; IBM Corporation, Armonk, NY, USA). Values are presented as the mean ± SD of at least three independent biological replicates. The significance of variations between mean values was statistically validated using Duncan’s multiple range test.

## 3. Results

### 3.1. 16S rDNA Sequencing Analysis of A. tumefaciens CL-1

**Figure 1:**
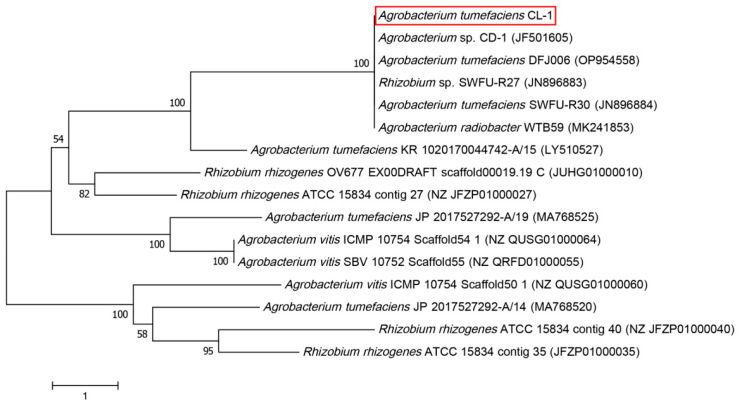
Phylogenetic tree of *Agrobacterium tumefaciens* CL-1. The tree was constructed using MEGA 11 software with the neighbor-joining method and 1000 bootstrap replicates. The accession numbers of reference strains are shown in parentheses.

The 16S rDNA amplified sequences were subjected to BLAST alignment on NCBI, revealing a large number of *A. tumefaciens* strains with over 99% sequence similarity to the studied strain *A. tumefaciens* CL-1. Some sequences were selected for phylogenetic tree analysis. The results showed that *A. tumefaciens* CL-1 clustered in the same branch with *Agrobacterium* sp. CD-1 (accession number: JF501605), *A. tumefaciens* DFJ006 (accession number: OP954558), *Rhizobium* sp. SWFU-27 (JN896883), *A. tumefaciens* SWFU-R30 (accession number: JN896884), and *A. radiobacter* WTB59 (accession number: MK241853) (Figure 1).

### 3.2. Isolation and Identification of Phage PAT-A

**Figure 2:**
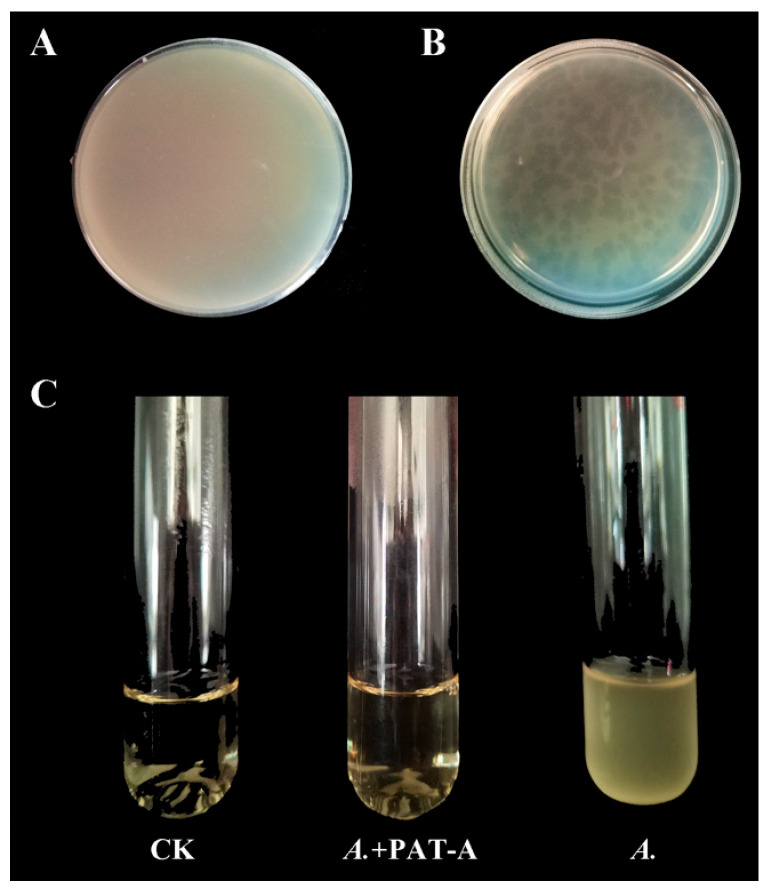
Plaque morphology characteristics and lytic effect of phage PAT-A. (**A**) Bacterial lawn of *Agrobacterium tumefaciens* CL-1 on double-layer agar plate; (**B**) Plaque morphology of phage PAT-A on double-layer agar plate; (**C**) Lytic effect after co-cultivation of phage PAT-A and *A. tumefaciens* CL-1. CK: LB only; *A.*+PAT-A: co-culture of *A. tumefaciens* CL-1 and phage PAT-A; *A.* only: *A. tumefaciens* CL-1 only.

As the control group, *A. tumefaciens* strain CL-1 was cultivated via the double-layer agar plate method. The results indicated that the plate was fully covered with bacteria (Figure 2A). In contrast, when soil sample filtrate was mixed with *A. tumefaciens* CL-1 and subjected to purification through the same method, distinct phage lysis plaques emerged after 24 h incubation at 28 °C. These circular, large, transparent plaques with well-defined edges exhibited characteristic features of lytic phages (Figure 2B). The phage lysis effect was further demonstrated through 6 h LB medium cultivation. Results showed that the CK group (without added strains or phages) remained clear and transparent. After co-cultivation with purified phages mixed with *A. tumefaciens* CL-1, the mixture was nearly transparent. In contrast, test tubes containing only *A. tumefaciens* CL-1 showed complete turbidity (Figure 2C).

**Figure 3:**
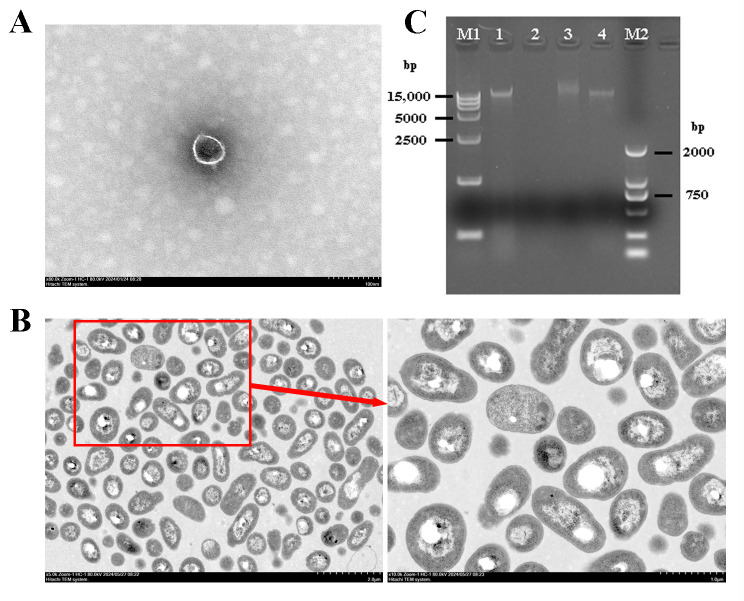
Transmission electron microscopic (TEM) images and nucleic acid type of phage PAT-A. (**A**) TEM image of phage PAT-A at 80 K magnification; (**B**) TEM images of *A. tumefaciens* CL-1 cells treated with phage at 5 K and 10 K magnifications; (**C**) Nucleic acid type identification of phage PAT-A. M1: DNA marker 15,000; 1: Control (PAT-A DNA); 2: Treatment with DNase I; 3: Treatment with RNase A; 4: Treatment with Mung Bean Nuclease; M2: DNA marker 2000.

TEM observations showed that PAT-A was tadpole shaped, consisting of a head and tail. The head diameter was about 60 nm, and the tail measurement was approximately 30 nm (Figure 3A). TEM analysis showed that only a very small number of cells in the field of view had intact membrane structures. After 8 h of treatment with phage PAT-A, the pathogenic bacterial cells were severely damaged, and the shape of the cell membrane was severely altered. The phenomenon of cytoplasmic wall separation was obvious, and voids appeared inside the cells, even forming a hollow cell skeleton, ultimately leading to cell lysis and death (Figure 3B).

The phage PAT-A genome was treated with DNase I, RNase A, and Mung Bean Nuclease. The results of nucleic acid electrophoresis showed that the genome of phage PAT-A could be completely degraded by DNase I, and there was no significant change after treatment with RNase A, indicating that its genome nucleic acid was DNA. Additionally, since the nucleic acid could not be degraded by Mung Bean Nuclease, it indicated that the phage genome was double-stranded DNA (dsDNA) (Figure 3C).

### 3.3. Biological Characteristics of Phage PAT-A

*A. tumefaciens* CL-1 and phage PAT-A were mixed in corresponding proportions. Following an 8 h cultivation period, the titer of bacteriophages was determined (Table 1). The findings indicated that when MOI was 0.01, the supernatant after cultivation exhibited the highest titer of bacteriophages, suggesting that the optimal multiplicity of infection for phage PAT-A was 0.01.

**Figure 4:**
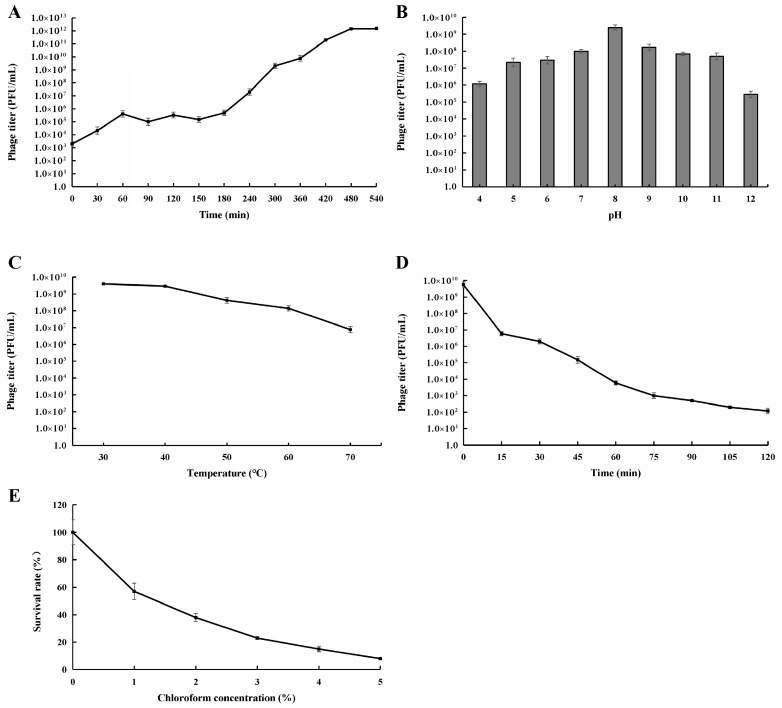
Biological characteristics of phage PAT-A. (**A**) One-step growth curve; (**B**) Phage infectivity after incubation at different pHs for 60 min. (**C**) Phage titer after being treated with different temperatures for 60 min. (**D**) Phage titer after being irradiated under an ultraviolet lamp (30 W) at different times. (**E**) Phage titer after being treated with chloroform in different ratios. Error bars indicate standard deviations of means (*n* = 3).

The one-step growth curve of phage PAT-A and *A. tumefaciens* CL-1 was ascertained by mixing them at the optimal multiplicity of infection of 0.01. Phage PAT-A exhibited a relatively long latent period, approximately 180 min, and a relatively long burst period, around 300 min (Figure 4A). Through calculation, the phage lytic titer was approximately 417 PFU cell^−1^, suggesting that phage PAT-A possessed a highly potent lytic and replicative capacity. Phage PAT-A maintained favorable activity within the pH range of 5.0 to 11.0, and its titer reached the maximum at pH 8.0. As the pH value either increased or decreased, the titer of the bacteriophage displayed a downward tendency (Figure 4B), indicating that phage PAT-A could endure weak acid and weak alkaline conditions, demonstrating excellent acid-base resistance. Phage PAT-A demonstrated good activity within the temperature range of 30 °C to 70 °C, and there was a downward trend with the increase in temperature (Figure 4C), suggesting that phage PAT-A had high thermal stability. After 15 min of UV irradiation, the titer of the bacteriophage decreased precipitously. As time elapsed, the rate of decrease in survival became slower. After 120 min of treatment, there were still 1.2 × 10^2^ PFU mL^−1^ of bacteriophage surviving (Figure 4D), indicating that phage PAT-A was relatively sensitive to UV but also had a certain degree of UV resistance. Chloroform had a significant impact on the titer of bacteriophages, with a sharp decline in the survival rate as the chloroform concentration increased (Figure 4E). At a chloroform concentration of 5%, the survival rate of bacteriophages was merely 8%, indicating that phage PAT-A was sensitive to chloroform.

### 3.4. Genomic and Phylogenetic Analyses of PAT-A

**Figure 5:**
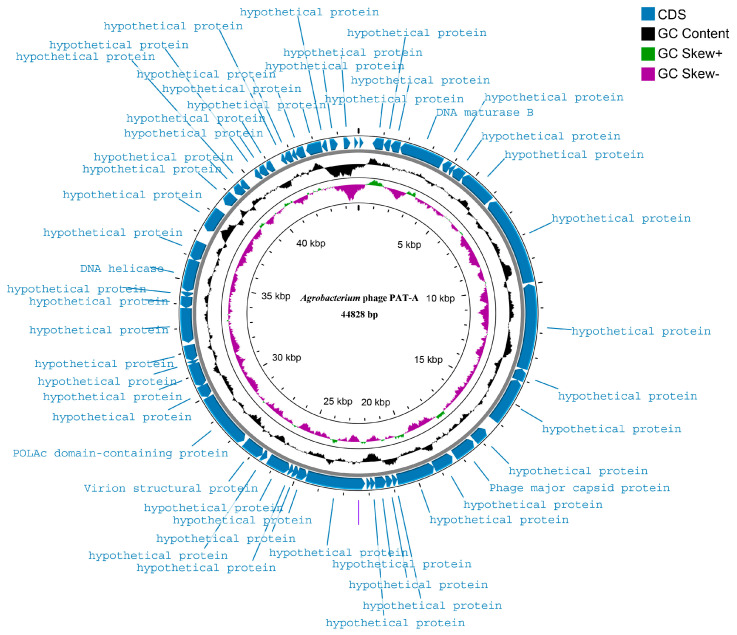
Genomic circular map of phage PAT-A. The outermost ring represents the 55 coding sequences encoded by the genome, with specific functions annotated in text; the next outer ring represents the GC skew of the genome; the next inner ring represents the GC content of the genome; and the innermost ring represents the PAT-A genomic position scale.

In this study, the genome of the PAT-A was linear, and a circular map analysis was performed for the genome 44,828 bp in length (Figure 5). As observed from the CDS annotations (blue) on the outer circle of the map, the coding sequences (CDSs) were almost continuously distributed along the genome with minimal intervals, exhibiting the typical compact organizational feature common to phage genomes. Regarding functional annotation, the majority of genes encoded hypothetical proteins, with only a few functionally annotated genes scattered throughout the genome. Examples included the annotated DNA helicase, POLA_c domain-containing protein, DNA maturase B, as well as structure-related genes such as Virion structural protein and Phage major capsid protein (Figure 5). The inner circle represented the GC content (black curve), which showed little fluctuation overall, indicating that the GC content of the genome was relatively stable across its entire length. In contrast, the GC skew (green for positive skew and purple for negative skew) displayed distinct segmental variations, with obvious positive-negative skew transitions occurring at approximately 5 kb and 25 kb (Figure 5). Combined with the annotated positions of replication/nucleic acid metabolism-related genes on the map, these skew transition regions can serve as references for further identifying potential key replication-related regions in subsequent studies. No integrase or lysogenic related repressor genes were detected in the genome, while the lytic module was intact, suggesting that this bacteriophage may be a strictly lytic type.

**Figure 6:**
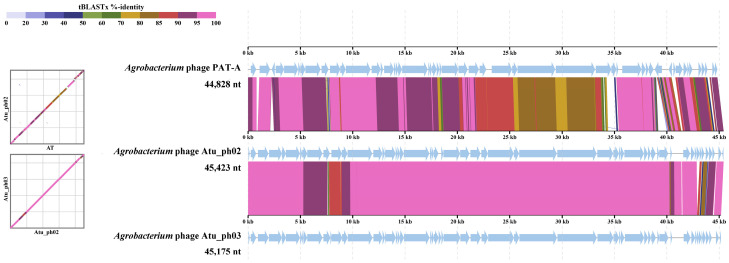
Genome comparison of phage PAT-A and its close homologues. The colored vertical blocks between genomes represent the degree of nucleotide similarity. The genome alignment was generated using ViPTree. The arrows in the figure indicate the transcriptional direction of coding sequences (CDSs) in the phage genomes.

To evaluate the taxonomic status and genomic structural characteristics of the phage PAT-A, whole-genome synteny analysis was performed using the tBLASTx algorithm (as part of the BLAST software suite, version 2.15.0; accessed via the official NCBI BLAST website: https://blast.ncbi.nlm.nih.gov/Blast.cgi, on 29 December 2025) between phage PAT-A and the two phages with the highest homology in the database—*Agrobacterium* phage Atu_ph02 (NC_047845) and *Agrobacterium* phage Atu_ph03 (NC_047846) (Figure 6). Comparative genomics analysis revealed that the full-length genome of phage PAT-A was 44,828 bp, which was highly similar in size to Atu_ph02 (45,423 bp) and Atu_ph03 (45,175 bp). As observed from the tBLASTx heatmap, phage PAT-A exhibited extremely high sequence identity with these two reference phages across the entire genome. The large areas of purple and deep red connecting regions in the figure indicated that the nucleotide sequence identity of the vast majority of coding sequences (CDSs) exceeded 90% and even reached over 95%. Furthermore, the synteny map (scatter plot on the left and linear alignment on the right of the figure) revealed a highly conserved genomic architecture among the three phages. A strict linear correspondence existed between phage PAT-A and the reference genomes, with no significant large-scale inversions or translocations detected. The gene order (synteny) and transcriptional direction were basically consistent across the three genomes. Although minor variations were present in certain regions (e.g., the middle and terminal parts of the genome, indicated by brown or yellowish-green bands in the figure), suggesting potential small-scale sequence differences or gene insertions/deletions in these regions, the overall genomic backbone remained highly stable. In summary, phage PAT-A shared an extremely close phylogenetic relationship with *Agrobacterium* phage Atu_ph02 and Atu_ph03, phylogenetically belonged to the same genus or even the same species, and shared a set of conserved core genomic backbones.

**Figure 7:**
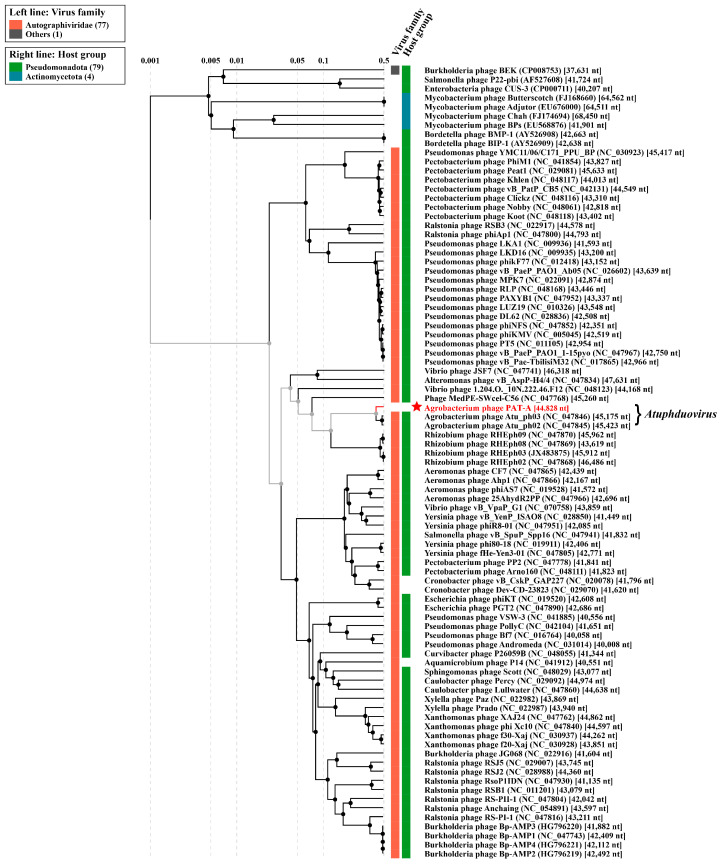
Proteomic tree of phage PAT-A. The PAT-A proteomic tree generated by ViPTree based on tBLASTx calculation of whole genome sequence similarity shows the distribution of PAT-A among the species within the genus *Atuphduvirus* (family Autographiviridae).

Furthermore, this study constructed a proteomic tree of the PAT-A genome sequence and identified closely homologous and outliers based on whole-genome sequence similarity calculated using tBLASTx. The results showed that PAT-A in *Atuphduvirus* of the Autographiviridae family formed a clade with *Agrobacterium* phage Atu_ph02 and *Agrobacterium* phage Atu_ph03 (Figure 7). Therefore, the AT genome was considered a provisional new member of *Atuphduvirus*.

### 3.5. The Efficacy of Phage PAT-A in Controlling Crown Gall Disease

**Figure 8:**
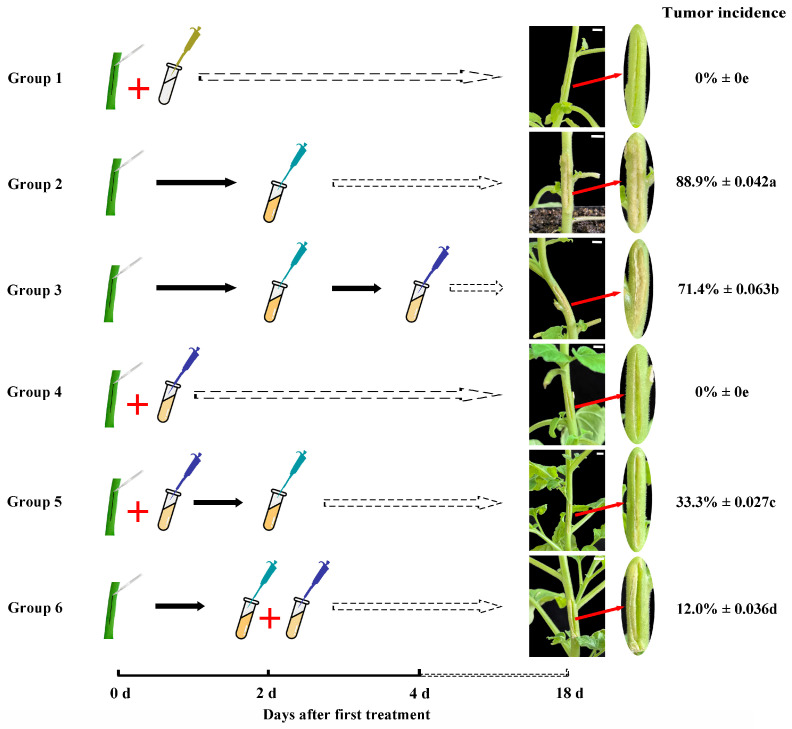
The biocontrol effect of phage PAT-A on tobacco plants. Group 1: negative control; Group 2: positive control; Group 3: inoculation with phage PAT-A after 2 days of *A. tumefaciens* CL-1 infection; Group 4: phage PAT-A treatment alone; Group 5: inoculation with phage PAT-A 2 days before *A. tumefaciens* CL-1 infection; Group 6: inoculation with both phage PAT-A and *A. tumefaciens* CL-1. Values are presented as the mean ± SD of at least three independent biological replicates. The significance of variations between mean values was statistically validated using Duncan’s multiple range test. Lowercase letters a–e indicate significant differences among groups (*p* < 0.05). The white indicator bar in the figure represents a scale bar of 1 cm. The black solid arrow denotes a 2-day time period, while the dashed arrow represents the time period from treatment completion to the 18th day (when the tumor incidence was counted).

To clarify the efficacy of phage PAT-A in controlling crown gall disease mediated by *A. tumefaciens*, this study set up six treatment groups according to the aforementioned experimental design. After artificially creating wounds on the stems of tobacco plants, inoculation treatments were carried out according to the set plans for each group. Eighteen days after infection, the formation of tumors at the wound sites was observed and the incidence rate was calculated. The results showed that: Group 1 (negative control) and Group 4 (phage PAT-A treatment alone) did not show any tumor formation on the tobacco wounds, with an infection rate of 0%; Group 2 (positive control), which was inoculated with *A. tumefaciens* CL-1 alone, had the highest incidence rate at 88.9% (±0.042); Group 3 (inoculation with phage PAT-A after 2 days of *A. tumefaciens* CL-1 infection) had a lower incidence rate than Group 2, at 71.4% (±0.063); Group 5 (inoculation with phage PAT-A 2 days before *A. tumefaciens* CL-1 infection) could effectively reduce the incidence rate to 33.3% (±0.027); Group 6 (optimized treatment group, inoculated with both phage PAT-A and *A. tumefaciens* CL-1) had the lowest incidence rate, at only 12.0% (±0.036) (Figure 8). It was worth noting that the incidence of crown gall disease in different treatment groups showed a positive correlation with the severity of tumors: the group with higher incidence (such as Group 2, Group 3) had more severe tumor symptoms on their plants; conversely, the group with lower incidence (such as Group 5, Group 6) had relatively mild tumor development.

## 4. Discussion

Bacteriophages are potent and efficient biocontrol agents. They can replicate at the infection site, reducing repeated applications and ensuring sustained antimicrobial efficacy [22,23]. Unlike other biocontrol agents, bacteriophages can co-evolve with bacterial populations, overcoming resistance and ensuring long-term effectiveness of biological control [24]. However, there is a shortage of effective lytic bacteriophages against *A. tumefaciens*, a cause of crown gall disease in plants, which is a key bottleneck in controlling this disease biologically [25]. This study successfully isolated and identified a new lytic bacteriophage PAT-A, which can specifically target the pathogen *A. tumefaciens* CL-1 associated with cherry tree crown gall disease, and its biocontrol potential has been systematically verified.

Morphologically, PAT-A exhibited a typical tadpole shape (60 nm head diameter, 30 nm tail length), a characteristic shared by many phages of the *Autographiviridae* family [25,26]. TEM observations further confirmed its strong lytic activity: after 10 h of co-cultivation with *A. tumefaciens* CL-1, the bacterial cell membrane was severely damaged, cytoplasmic wall separation occurred, and hollow cell skeletons formed, which was consistent with the lytic mechanism of phages that disrupted bacterial structural integrity and metabolic processes [25]. Nucleic acid analysis identified PAT-A as a double-stranded DNA (dsDNA) phage, a common feature among phages with high stability and replicative capacity, which was favorable for its potential formulation as a biocontrol agent [27,28].

In terms of biological characteristics, PAT-A exhibited favorable environmental adaptability. The optimal multiplicity of infection (MOI) is a major indicator of their infectivity and can be used for subsequent large-scale production of bacteriophages [29]. The MOI of 0.01 and the phage lytic titer (417 PFU cell^−1^) indicated that PAT-A can efficiently replicate and lyse host bacteria at low concentrations, reducing the cost of large-scale application. PAT-A could withstand a wide range of pH and temperature fluctuations, indicating that it was quite stable in the soil and could maintain infectivity for a long time [30]. The detrimental impacts of UV radiation on microbes have been extensively recognized and explored in studies on microbial ecology [31]. Long-term UV irradiation significantly affected the phage titer, consistent with previous studies [32]. Although PAT-A was sensitive to UV radiation (titer declining sharply after 15 min) and chloroform (8% survival at 5% concentration), these limitations could be mitigated through formulation strategies (e.g., adding UV protectants or encapsulation) [33].

Genomically, PAT-A had a 44,828 bp genome encoding 55 open reading frames (ORFs) with a compact structure. The absence of integrase or lysogenic repressor genes, combined with the intact lytic module (including peptidoglycan hydrolase and cell wall hydrolase), confirmed its strictly lytic nature. This was a critical advantage over lysogenic phages, which may transfer harmful genes (e.g., antibiotic resistance genes) to host bacteria or enter a dormant state, limiting their biocontrol efficacy [28]. Phylogenetic analysis revealed that PAT-A clustered with *Agrobacterium* phages Atu_ph02 and *Agrobacterium* phages Atu_ph03 in the *Atuphduvirus* genus of Autographiviridae, with high genomic collinearity [25]. This suggested evolutionary conservation within the genus while highlighting PAT-A’s uniqueness, as structural variations in central and terminal genomic regions may contribute to its specific host range and adaptability—key factors for targeting *A. tumefaciens* CL-1. The narrow host range of PAT-A conferred unique application advantages. Given that phages lack infectivity against non-target beneficial bacteria, they can precisely target pathogenic strains during crown gall disease control, avoiding disruption of soil microbial balance. However, its high specificity for limited *A. tumefaciens* strains poses a critical bottleneck in practical applications [25]. Under natural conditions, the limited host spectrum of PAT-A may hinder coverage of diverse field pathogen populations, thereby affecting the stability and universality of control efficacy.

Most importantly, the biocontrol experiment demonstrated PAT-A’s significant efficacy against crown gall disease in tobacco. The Group 6 treatment (simultaneous inoculation of PAT-A and *A. tumefaciens* CL-1) achieved the lowest tumor incidence (12.0%), followed by pre-inoculation of PAT-A (Group 5: 33.3% incidence). In contrast, post-inoculation (Group 3: 71.4% incidence) was less effective, and the positive control (Group 2: 88.9% incidence) confirmed the virulence of *A. tumefaciens* CL-1. However, Group 6 was too idealized. In actual production, the simultaneous infection of bacteriophages and pathogenic bacteria occurred with very low probability. Therefore, this result emphasized the importance of timing: pre-application of PAT-A prevents bacterial colonization at wound sites (the primary entry point for *A. tumefaciens*), whereas post-application may fail to eliminate established bacteria that have already initiated Ti plasmid transfer. This finding was consistent with previous studies on phage biocontrol of bacterial diseases, where early intervention was critical for suppressing pathogen proliferation [34,35,36]. Additionally, the absence of tumor formation in Group 4 (PAT-A alone) confirmed PAT-A’s safety for host plants, addressing concerns about non-target effects, a key advantage over chemical bactericides.

## 5. Conclusions

This study isolated and characterized a novel strictly lytic phage PAT-A, which belonged to the *Atuphduvirus* genus of Autographiviridae. PAT-A exhibited favorable biological characteristics (broad pH/temperature tolerance, high burst size, optimal MOI of 0.01) and significant biocontrol efficacy against crown gall disease in tobacco. Genomic analysis confirmed its lytic nature and evolutionary conservation within Atuphduvirus, while morphological and nucleic acid studies validated its stability and replicative capacity. This research enriches the phage resource pool for crown gall disease control, provides novel candidate phages for its biocontrol, and delves into the molecular mechanisms of phage-based biocontrol strategies. It further highlights the significance of phage characterization (genomics, biology) and application timing in developing effective approaches, thereby offering critical theoretical insights and practical support for the implementation of phage-based biocontrol.

## Figures and Tables

**Table 1:** Optimal multiplicity of infection (MOI) of phage PAT-A.

Multiplicity of Infection (MOI)	Phage Concentration (PFU mL^−1^)	Bacteria Concentration (CFU mL^−1^)	Phage Titer (PFU mL^−1^)
0.001	10^6^	10^9^	2.95 × 10^12^
0.01	10^7^	10^9^	4.50 × 10^12^
0.1	10^8^	10^9^	9.00 × 10^11^
1	10^9^	10^9^	6.75 × 10^11^
10	10^10^	10^9^	6.60 × 10^11^
100	10^11^	10^9^	1.10 × 10^10^

## Data Availability

The original contributions presented in the study are included in the article, further inquiries can be directed to the corresponding author.
